# Influence of Pore Structure and Metal‐Node Geometry on the Polymerization of Ethylene over Cr‐Based Metal–Organic Frameworks

**DOI:** 10.1002/chem.202005308

**Published:** 2021-03-01

**Authors:** Maarten K. Jongkind, Miguel Rivera‐Torrente, Nikolaos Nikolopoulos, Bert M. Weckhuysen

**Affiliations:** ^1^ Inorganic Chemistry and Catalysis Debye Institute for Nanomaterial Science Utrecht University Universiteitsweg 99 3584 CG Utrecht The Netherlands

**Keywords:** catalyst characterization, chromium, ethylene polymerization, metal–organic frameworks

## Abstract

Metal–organic frameworks (MOFs) have received increasing interest as solid single‐site catalysts, owing to their tunable pore architecture and metal node geometry. The ability to exploit these modulators makes them prominent candidates for producing polyethylene (PE) materials with narrow dispersity index (*Ð*) values. Here a study is presented in which the ethylene polymerization properties, with Et_2_AlCl as activator, of three renowned Cr‐based MOFs, MIL‐101(Cr)‐NDC (NDC=2,6‐dicarboxynapthalene), MIL‐53(Cr) and HKUST‐1(Cr), are systematically investigated. Ethylene polymerization reactions revealed varying catalytic activities, with MIL‐101(Cr)‐NDC and MIL‐53(Cr) being significantly more active than HKUST‐1(Cr). Analysis of the PE products revealed large *Ð* values, demonstrating that polymerization occurs over a multitude of active Cr centers rather than a singular type of Cr site. Spectroscopic experiments, in the form of powder X‐ray diffraction (pXRD), UV/Vis‐NIR diffuse reflectance spectroscopy (DRS) and CO probe molecule Fourier transform infrared (FTIR) spectroscopy corroborated these findings, indicating that indeed for each MOF unique active sites are generated, however without alteration of the original oxidation state. Furthermore, the pXRD experiments indicated that one major prerequisite for catalytic activity was the degree of MOF activation by the Et_2_AlCl co‐catalyst, with the more active materials portraying a larger degree of activation.

## Graphical abstract

**Multiple active sites**: MIL‐101(Cr)‐NDC, MIL‐53(Cr) and HKUST‐1(Cr) were tested for ethylene polymerization and found to produce different polyethylene (PE) products and PE morphologies. Analysis of the PE revealed that multiple active sites exist, indicating that the active phase cannot be considered a single‐site catalyst. An array of spectroscopic techniques was used to investigate active site formation for the aforementioned metal–organic frameworks (MOFs).

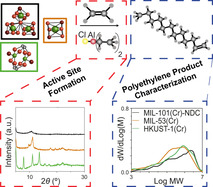

## Introduction

The production processes of plastics, of which polyethylene has the largest market volume share, stands as one of the most mature, sustainable, and efficient technologies relying on fossil and, more recently, renewable feedstocks. Despite increased attention to the environmental issues that plastics cause, the broad range of applications ensures that the production of PE will continue to grow in the coming years.^[1]^ These factors continue to drive research towards improved production of PE, as well as towards finding new types of PE.

Nowadays, polyethylene production is centered around three catalytic workhorses, namely Ziegler–Natta, (post‐)metallocenes and Cr/SiO_2_ Phillips type catalysts, each catalyst producing a variety of PEs in terms of molecular weight distribution (MWD), short‐chain, and, long‐chain branching (SCB and LCB).[^2^, ^3^, ^4^, ^5^, ^6^] In addition to selecting the proper catalyst, reaction conditions and reactor configurations can be exploited to enhance or suppress specific PE properties.[^7^, ^8^, ^9^, ^10^, ^11^] However, as mentioned before, the discovery and development of new catalysts keeps driving worldwide research efforts.

Metal–organic frameworks (MOFs) are defined by a high degree of ordering in terms of pore size and pore structure, as well as the possibility to exploit a wide variety of metal‐sites, tailoring their activity by rational design.[^12^, ^13^, ^14^, ^15^, ^16^, ^17^] This has been elegantly exemplified by the linear correlation between catalytic CO_2_ photoreduction, as a model reaction, and the electronic structure of the linker, showing that functional groups on the organic linker directly affect the reactivity.[^15^, ^16^, ^17^, ^18^, ^19^, ^20^] This method is comparable to conventional strategies for heterogenous catalyst design, such as using dopants (promoters) or tuning the support,[^3^, ^21^, ^22^, ^23^, ^24^, ^25^, ^26^] but with the added value of a straightforward characterization and understanding of the material. The ability to change the pore structure, metal‐node and electronic structure with relative ease, make MOFs increasingly prominent candidates for heterogenized single‐site catalysis.[^27^, ^28^, ^29^] Until now, they have been used as such in numerous reactions, for example, others alkene oligomerization reactions[^30^, ^31^, ^32^, ^33^, ^34^, ^35^, ^36^, ^37^, ^38^, ^39^] as well as polymerization reactions.[^30^, ^37^, ^40^, ^41^, ^42^, ^43^, ^44^, ^45^, ^46^, ^47^, ^48^]

Dincǎ, Roman‐Leshkov and co‐workers investigated the ethylene polymerization properties of MFU‐41 (MFU=metal–organic framework Ulm‐University) MOFs.[^41^, ^45^] The gas‐phase ethylene polymerization properties of Cr^3+^ ion‐exchanged MFU‐4l MOF material were investigated, revealing a polymerization MOF that produced PE with a dispersity index (*Ð*) of about 1.4, the *Ð* being defined as the *M_w_/M_n_*, with the *M_w_* representing the weight averaged molecular weight of the polymer and the *M_n_* representing the number averaged molecular weight of the polymer. This indicates that active sites are similar in nature, and is an excellent example of a Cr‐based MOF acting as a heterogenized single‐site ethylene polymerization catalyst. However, in the case of MOFs, forming true single‐site catalysts depends strongly on the material structure and its evolution upon reaction with co‐catalyst species; and it merits detailed studies on a per case basis. Previous work from our group compared the polymerization properties of MIL‐101(Cr) vs. MIL‐100(Cr) and revealed that the type of linker is of paramount importance for the ability, or inability, of the MOF to fragment. Here, the ability of the MOF (MIL‐101(Cr)) to fragment was correlated to the ability to polymerize ethylene, whereas the inability to fragment (MIL‐100(Cr)) was related to predominantly α‐oligomerization and negligible polymerization activities.[^35^, ^43^]

Despite all these advantages and already numerous applications, one should be cautious in using Cr‐based catalysts due to their toxicity as well as related environmental hazards. Fortunately, this is of no real concern in olefin polymerization, since state of the art catalysts portray such high activities that noncorrosive and nontoxic catalyst residues can be safely left in the PE product material in sub‐ppb levels.[^49^, ^50^]

**Figure 1:**
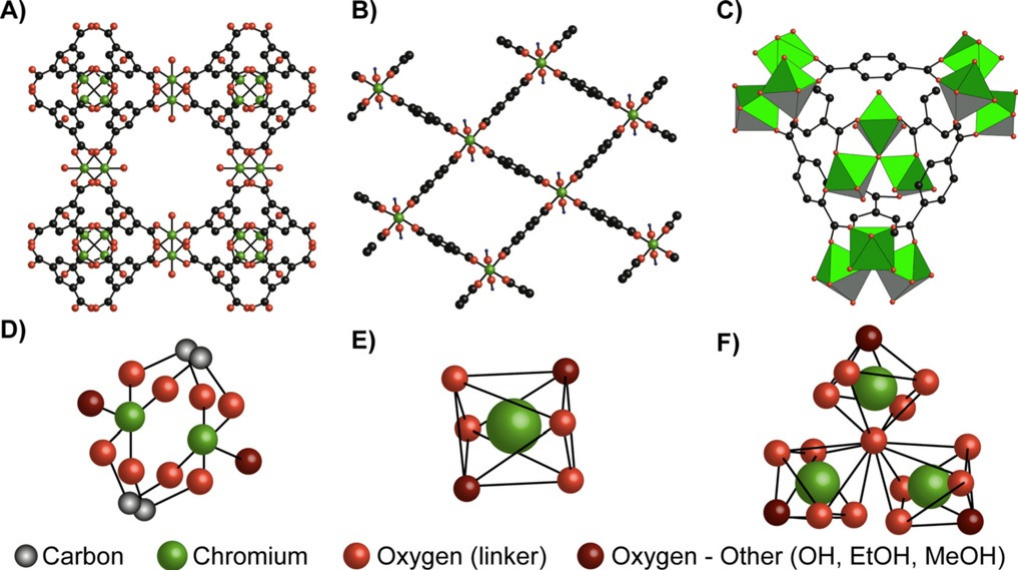
A) Overview of the HKUST‐1(Cr) pore geometry and unit cell. B) Overview of the MIL‐53(Cr) pore geometry and unit cell. C) Overview of the MIL‐101(Cr) pore geometry and unit cell. D) Metal‐node structure of HKUST‐1(Cr). E) Metal‐node structure of MIL‐53(Cr). F) Metal‐node structure of MIL‐101(Cr).

In view of an increasing interest towards the application of MOFs in ethylene polymerization, we opted to investigate several well renowned Cr‐based MOFs in this reaction, their structures demonstrated in Figure 1, specifically: MIL‐101(Cr)‐NDC (NDC=2,6‐dicarboxynapthalene),[^17^, ^51^, ^52^, ^53^, ^54^] MIL‐53(Cr)[^55^, ^56^, ^57^] and HKUST‐1(Cr).^[58]^ In order to establish structure–activity relationships, this selection of MOFs comprises a variety of pore sizes, 3D‐structures and metal‐node geometries. We investigated the fate of these materials when reacting with Et_2_AlCl to activate the MOF towards ethylene polymerization in the slurry‐phase. Two different hydrocarbon solvents, heptane and toluene, were studied as reaction media. The molecular architecture and morphology of the resulting polymers were, respectively investigated with gel permeation chromatography (GPC), differential scanning calorimetry (DSC), and with scanning electron microscopy (SEM). Moreover, we sought to understand the underlying mechanisms of how Et_2_AlCl activates the MOF in terms of crystallinity, surface area, Cr oxidation state and surface site accessibility, by means of a broad array of tools including X‐ray Diffraction (XRD), UV/Vis‐NIR diffuse reflectance spectroscopy (DRS), CO probe molecule Fourier transform infrared (FTIR), N_2_ physisorption, diffuse reflectance infrared spectroscopy (DRIFTS) and scanning electron microscopy (SEM).

## Results and Discussion

The MOFs under investigation, MIL‐101(Cr)‐NDC,^[51]^ MIL‐53(Cr)^[55]^ and HKUST‐1(Cr)^[58]^ were prepared in accordance to already reported procedures and their thermal stability was assessed by means of TGA as reported in Supporting Information, sections 1 and 2.

### Ethylene polymerization reactions

Table 1 shows the activity in ethylene polymerization of the different MOFs in both heptane and toluene as diluents. It is well known that the selected solvent can detrimentally affect ethylene polymerization/oligomerization properties, either due to coordination to the active site or decomposition of the active site.[^59^, ^60^]

**Table 1:** Properties of the polyethylene properties produced with the heterogeneous reactions and the filtrate reactions.

	MOF activity [kg_PE_ mol_Cr_^−1^ h^−1^ bar^−1^]		
	MIL‐101(Cr)‐NDC	MIL‐53(Cr)	HKUST‐1(Cr)
heptane	1.24	4.01	0.22
filtrate^[a]^	0.08	1.19	0.089
% activity from leached Cr species	6.5	29.7	40.5
no activator	*inactive*	*inactive*	*inactive*
toluene	0.87	0.39	0.11

Reactions conditions unless specified otherwise: 10 bar C_2_H_4_, *T*=25 °C, [Cr]=5×10^−2^  mmol was used in 15 mL solvent with mol [Al/[Cr]=100 and Et_2_AlCl. [a] A reaction mixture of the filtrate is produced by reacting [Cr]=5×10^−2^ mmol MOF with 100 mol. eq. Et_2_AlCl in 10 mL heptane. Then, the filtrate is collected, and the filter is washed twice with 2 mL heptane and once with 1 mL heptane, to add the leached Cr into the solution.

All three MOF structures showed higher productivity in heptane, likely due to the absence of potential Cr^3+^–π ring interactions which may be present when toluene is used.[^59^, ^60^, ^61^] The catalytic activities in Table 1 confirm that selection of the proper MOF precursor is of paramount importance and the topology plays a dominant role in polymerization activity. MIL‐53(Cr) is the most active MOF, with an activity of 4.01 kg_PE_ mol_Cr_^−1^ h^−1^ bar^−1^, followed by MIL‐101(Cr)‐NDC with an activity of 1.24 kg_PE_ mol_Cr_^−1^ h ^−1^ bar^−1^, and HKUST‐1(Cr), exhibiting a poor activity of 0.22 kg_PE_ mol_Cr_^−1^ h ^−1^ bar^−1^. Table 1 also clearly demonstrates the importance of selecting the proper reaction medium, since MIL‐53(Cr) loses the majority of its activity upon switching to toluene. Similarly, HKUST‐1(Cr) lost about 50 % of its original activity and only MIL‐101(Cr)‐NDC appeared to be relatively unaffected by the change in reaction medium, retaining about 75 % of its original activity.

As already mentioned in the introduction: The current state of the art polyolefin catalysts portrays such activities that the catalyst residues can be harmlessly left in the PE material. The MOF residue percentages in the final PE products vary from 24 wt % for HKUST‐1(Cr) to 0.4 wt % for MIL‐53(Cr) in the here discussed polymerization reactions, which is not (yet) ideal. However, we were limited in terms of catalyst yields by the reaction vessel size. By simply increasing the reactor volume and increasing the reaction time, it is possible to increase the PE yield and lower related MOF contribution to the final product composition.

As shown in our previous efforts, Cr may partly leach from the MOFs into the solution.^[43]^ Hence, to investigate the contribution of such species to polymer formation, the reactions were also performed after filtration of the MOFs after reaction with the co‐catalyst, and labeled “filtrate reactions”. We found that for all the MOF topologies under study, residual activity was obtained from the liquid, indeed confirming that a certain fraction of polymer may be produced by species in solution instead of Cr sites on/in the MOF lattice. In the case of MIL‐101(Cr)‐NDC, an activity of 0.08 kg_PE_ mol_Cr_^−1^ h^−1^ bar^−1^ is obtained, corresponding to 6.6 % of its original activity, while the filtrates of MIL‐53(Cr) and HKUST‐(Cr) demonstrated higher activities with values of, respectively 29.7 % and 40.5 %.

Inductively coupled plasma‐atomic emission spectroscopy (ICP‐OES) was used to quantify the amount of Cr leached from the framework. These measurements revealed that for MIL‐101(Cr)‐NDC, MIL‐53(Cr) and HKUST‐1(Cr) respectively 1.02 %, 4.96 % and 0.71 % of the original Cr content leached into solution. However, these Cr species were responsible for a significant fraction of the polymer produced (6.5, 29.7 and 40.5 %, respectively). This shows that although the MOF materials are relatively stable (<5 % Cr leached) towards disintegration by the co‐catalyst, the homogeneous compounds produced by the pre‐treatment are very active in ethylene polymerization.

### Polymer analysis

**Figure 2:**
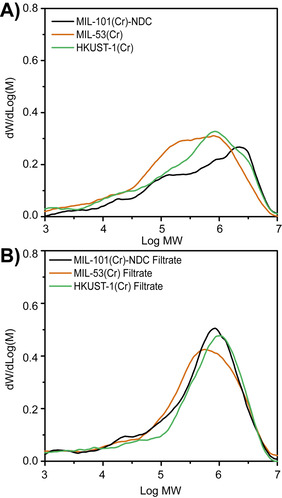
GPC traces of the polyethylene (PE) materials produced in the A) heterogeneous reactions and B) homogeneous reactions.

The results of the GPC analysis on the PE materials are shown in Table 2, with the corresponding traces of the PE materials produced in the heterogeneous reactions being shown in Figure 2 A and those produced in the homogeneous reactions demonstrated in Figure 2 B.

**Table 2:** Results of the ethylene polymerization reactions at 10 bar.

	*M_w_* ^[a]^ [kDa]	*M_n_* ^[a]^ [kDa]	*M_z_* ^[a]^ [kDa]	*Ð*	*T_m2_* [°C]^[b]^	*X_c2_* [%]^[b]^
Heterogeneous reactions						
MIL‐101(Cr)‐NDC	1100	39	3300	28.2	126.4	46.4
MIL‐53(Cr)	759	45	3592	16.9	133.8	52.4
HKUST‐1(Cr)	963	98	3466	9.8	133.8	39.3 (51.7)^[c]^

Filtrate reactions						
MIL‐101(Cr)‐NDC	922	146	3077	6.3	134.5	49.1
MIL‐53(Cr)	961	51	3467	18.8	134.2	57.0
HKUST‐1(Cr)	1102	337	3026	3.2	134.3	48.1

[a] Determined by GPC with PE and PP standards. [b] Determined by the melting enthalpy calculated from DSC in comparison to Δ*H* ^0^_m_=293 J g^−1^ for 100 % crystalline UHMWPE. [c] This value is the crystallinity after correcting for the significant catalyst residue contribution in the final PE/HKUST‐1(Cr) composition product.

The first observation are the broad MW curves in Figure 2, which coincidentally are also typical for high‐density polyethylene materials produced by (ill‐defined) Cr‐based Phillips‐type catalysts.[^3^, ^61^] For instance, both MIL‐101(Cr) and MIL‐53(Cr) demonstrate a variety of peaks in their respective GPC curves. On the other hand, the MWD curve for HKUST‐1(Cr) is a bit narrower, but still: multiple peaks are identifiable. These three curves gave rise to high *Ð* values of 28.2, 16.9 and 9.8 for MIL‐101(Cr)‐NDC, MIL‐53(Cr) and HKUST‐1(Cr), respectively. While these results do reflect a narrowing MWD, they are still far from values usually observed for single‐site catalysts, ultimately indicating the existence of a multitude of active sites on the MOFs, as was also the case for MIL‐101(Cr).^[43]^

The GPC curves, and related *Ð* values, are slightly different for the reactions performed with the filtrates. The traces of the PE obtained when using the Cr species filtered from MIL‐101(Cr)‐NDC and HKUST‐1(Cr) are significantly skewed around log MW≈6, suggesting that the number of active sites participating in polymer formation is smaller. This may be related to Cr species in solution, being Cr atoms with similar ligand coordination and no steric restrictions. Furthermore, and in contrast to the two other topologies, the *Ð* value obtained for the reaction performed with the filtrate of MIL‐53(Cr) is very similar to that for the reaction performed with MIL‐53(Cr) itself. Again, this finding shows that the number of Cr species generated from MIL‐53(Cr) is larger than in the other two cases.

Additionally, the differences in polymer architecture are further manifested in the different crystallinities, shown in Table 1. The resulting PE materials demonstrate varying crystallinities: the lowest being that of the PE produced with HKUST‐1(Cr) (39.3 %) and the highest being that of MIL‐53(Cr) (52.4 %). Interestingly, the PEs produced in the filtrate reactions all demonstrate higher crystallinities than their heterogeneous counterparts. Perhaps the fact that the average increased *M_n_* explains this, since this value infers an overall relative increase of average chain length, which can be associated to the higher crystallinity of the materials. Take note that the calculated crystallinity of the PE/HKUST‐1(Cr) material is underestimated due to the significant contribution of the HKUST‐1(Cr) MOF residue. The likely PE crystallinity is higher, and correcting for the MOF residue gives a value of 51.7 %.

Conclusively, it is evident from Table 1 and Figure 2 that rational selection of the MOF is a valid strategy for exposing and/or attaining desirable PE properties.

### Polyethylene morphology

Naturally, the PE morphology is highly important for assessing post‐reaction processability as well as preventing reactor fouling, often related to expensive reactor downtimes.^[62]^ From an industrial point of view, “good” is considered spherical with narrow particle size distributions, which is directly related to high bulk density, controlled porosity, controlled internal composition and high process flowability.[^63^, ^64^]

**Figure 3:**
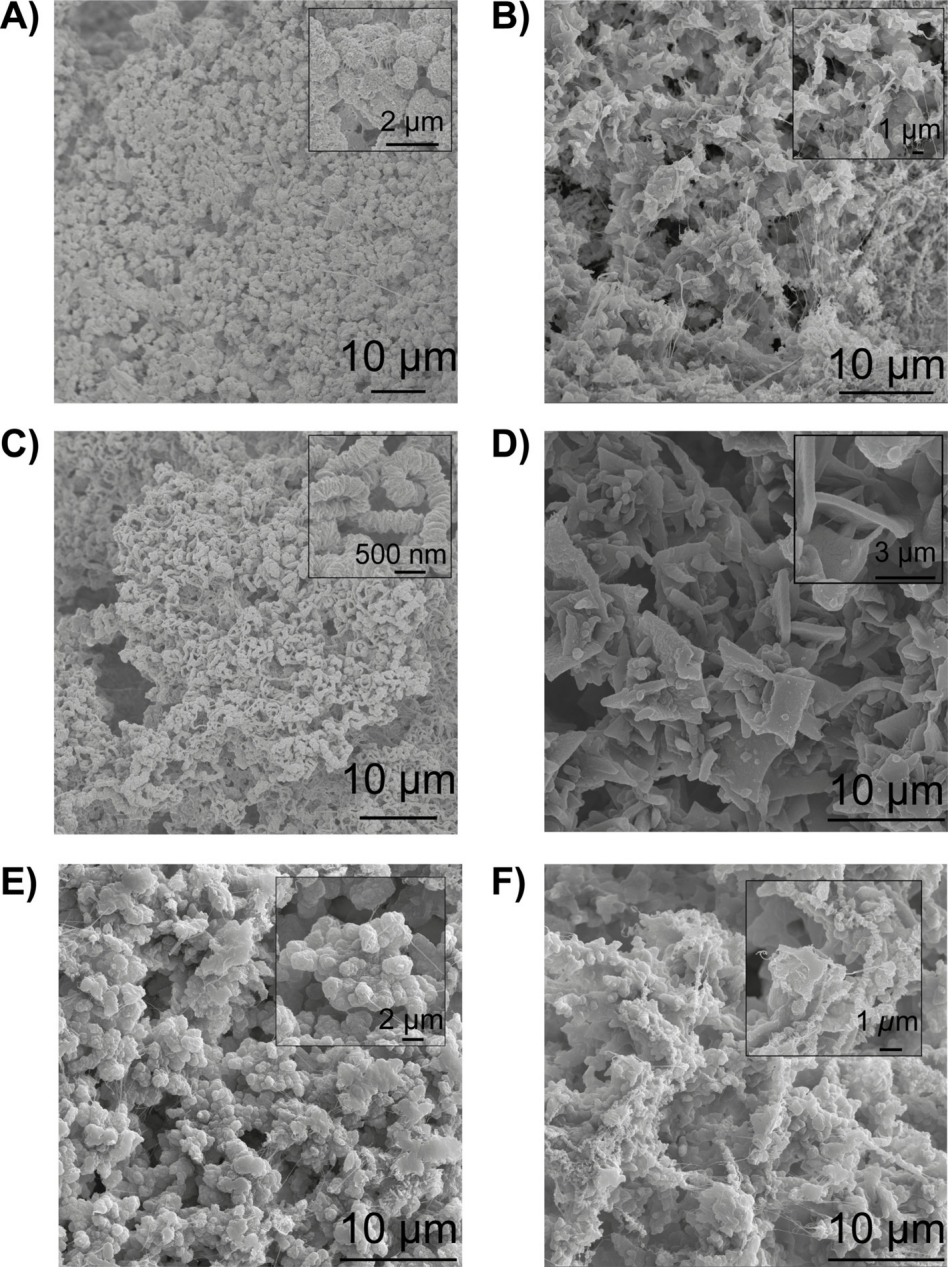
SEM images of the polyethylene (PE) materials produced with, respectively A) MIL‐101(Cr)‐NDC, B) filtrate from MIL‐101(Cr)‐NDC, C) MIL‐53(Cr), D) filtrate from MIL‐53(Cr), E) HKUST‐1(Cr) and F) the filtrate from HKUST‐1(Cr).

Figure 3 shows the obtained PE materials obtained from the ethylene polymerization reactions with the MOFs, and clearly demonstrates that one can affect the final PE morphology by selecting the proper MOF polymerization template, at least under the here described reaction conditions.

First, Figure 3 A shows that the PE produced by MIL‐101(Cr)‐NDC consists of spherical particles, where the inset shows sub‐crystallites being tied together by PE molecules. This indicates that the MOF acts as a self‐sacrificial template and disintegrates due to the increasing stress generated by the growing polyethylene. Despite this fragmentation, it seems that the originally octahedral MOF morphology enforces a spherical morphology on the PE. Secondly, the obtained morphology from the reactions with MIL‐53(Cr) is best described as a fibrous worm‐like PE material.^[65]^ Interestingly, Chanzy et al. attributed this mechanism of PE growth to active sites being in very close proximity, hereby hampering the PE growth in lateral directions while this is not the case perpendicular to the plane of active sites.^[65]^ Thus, this indicates that factors such as active site spacing are also an highly important parameter to consider when using MOFs in ethylene polymerization.

Despite the low activity of HKUST‐1(Cr), it mainly produced spherical PE beads, as shown in Figure 3 E, with some PE fibers as well. The dual morphology is likely explained by the fact that a significant percentage of the catalytic activity originates from homogeneous Cr sites that produce the fibrous PE material. Interestingly, the amount of fibrous material is relatively small and spherical particles are the predominant product. This indicates that even for the homogeneously polymerizing components, the MOF can act as the preferential growth template.

Figures 3 B, D and F are a testament to the importance of the MOF's role as structuring agent. In all instances, if this structuring agent is removed from the equation, a variety of structures (e.g. platelets, spheroids and fibers) is obtained. Irregularities on the reactor wall and/or stirrer now predominantly act as crystallization points for the (relatively small) waxes formed over the homogeneous Cr sites, after which PE continues growing.^[62]^

If anything, these SEM images highlight the importance of selecting the appropriate MOF as ethylene polymerization platform for attaining desirable PE morphologies. Additionally, and possibly more importantly, the simultaneous polymerization over heterogeneous Cr sites and homogeneous Cr sites is not detrimental for the final morphology, inferring that even for the dissolved active sites, and PE materials, the MOF crystallites act as crystallization point.

### Active site formation

The GPC results indicated the participation of a variety of active Cr sites in ethylene polymerization: both homogeneous and heterogeneous. Therefore, we opted to exploit an array of spectroscopic techniques such as XRD, CO probe molecule FTIR and UV/Vis‐NIR DRS experiments in order to investigate the MOF activation stage. Additionally, investigating the morphology of the pristine and activated MOFs required the use of SEM as an imaging technique.

**Figure 4:**
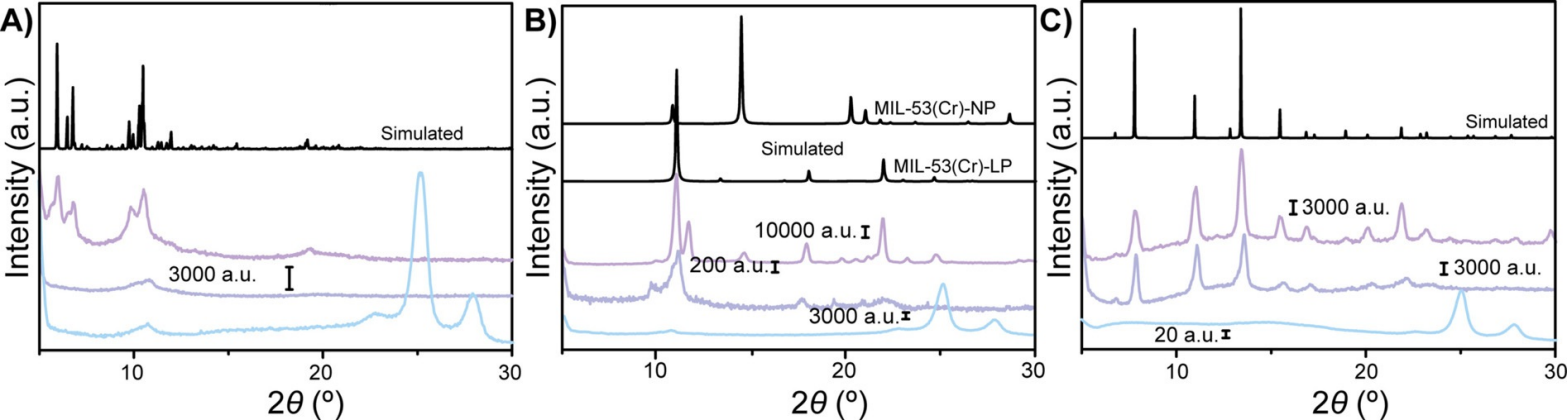
Results from the powder X‐ray diffraction (XRD) experiments for A) MIL‐101(Cr)‐NDC, B) MIL‐53(Cr) and C) HKUST‐1(Cr). Purple, top line, is the X‐ray diffractogram of the pristine MOF. Dark‐blue, middle‐line, is the *x*‐ray diffractogram of the MOF after activation with 100 mol. equiv. co‐catalyst. Light‐blue, bottom line, after the ethylene polymerization reaction.

First, XRD serves as an excellent tool to investigate the effect of the co‐catalyst on the MOF crystallinity, for which the results are shown in Figure 4. The X‐ray diffractograms of the three pristine MOFs perfectly resemble those from the literature, although noteworthy: The XRD pattern of MIL‐53(Cr) resembles that of the solvated MOF.[^51^, ^55^, ^58^] Now, the effect of Et_2_AlCl on the crystallinity of each MOF was different and will be discussed hereafter. If consumption of the MOF is to occur, this would coincide with a loss and/or broadening of the diffraction signals.

For both MIL‐101(Cr)‐NDC and MIL‐53(Cr) this indeed occurs, as demonstrated in Figures 4 A and B, respectively. Activation of MIL‐101(Cr) is related to a disappearance of the signals below diffraction angles of 10°. It can be argued that the diffraction signals at 10 can still be identified, albeit less intense, inferring that at least some of the crystallinity is retained. In Figure 4 B, activation of MIL‐53(Cr) is related to the disappearance of some of the diffraction peaks (most clearly the ones at 2*θ*=13, 15, 23 and 25°), indicating that the crystallinity of MIL‐53(Cr) is severely disrupted. It is worth stating that MIL‐53(Cr) is known to undergo structural transitions and exhibits a so‐called breathing effect, however it is highly unlikely that such events occur here, since only some of the typical XRD reflections for large pore (LP) structures is visible in this case.[^55^, ^66^, ^67^]

Interestingly, the crystallinity of HKUST‐1(Cr) is almost unaffected by the activation procedure with 100 mol. equiv. Et_2_AlCl, since the original diffraction pattern is largely retained. The decrease in intensity of diffraction peaks above 2*θ*=15° does infer some minor disruption of the crystallinity, however the severity is far from those found for MIL‐101(Cr)‐NDC and MIL‐53(Cr). This observation might provide part of the explanation for the low activity of HKUST‐1(Cr), since apparently this MOF is activated to the least extent.

Also, the X‐ray diffractograms are a testament to the polymerization of ethylene over the MOFs, since instead of MOF related reflections, only HDPE X‐ray diffraction peaks are observed, specifically at 23°.

**Figure 5:**
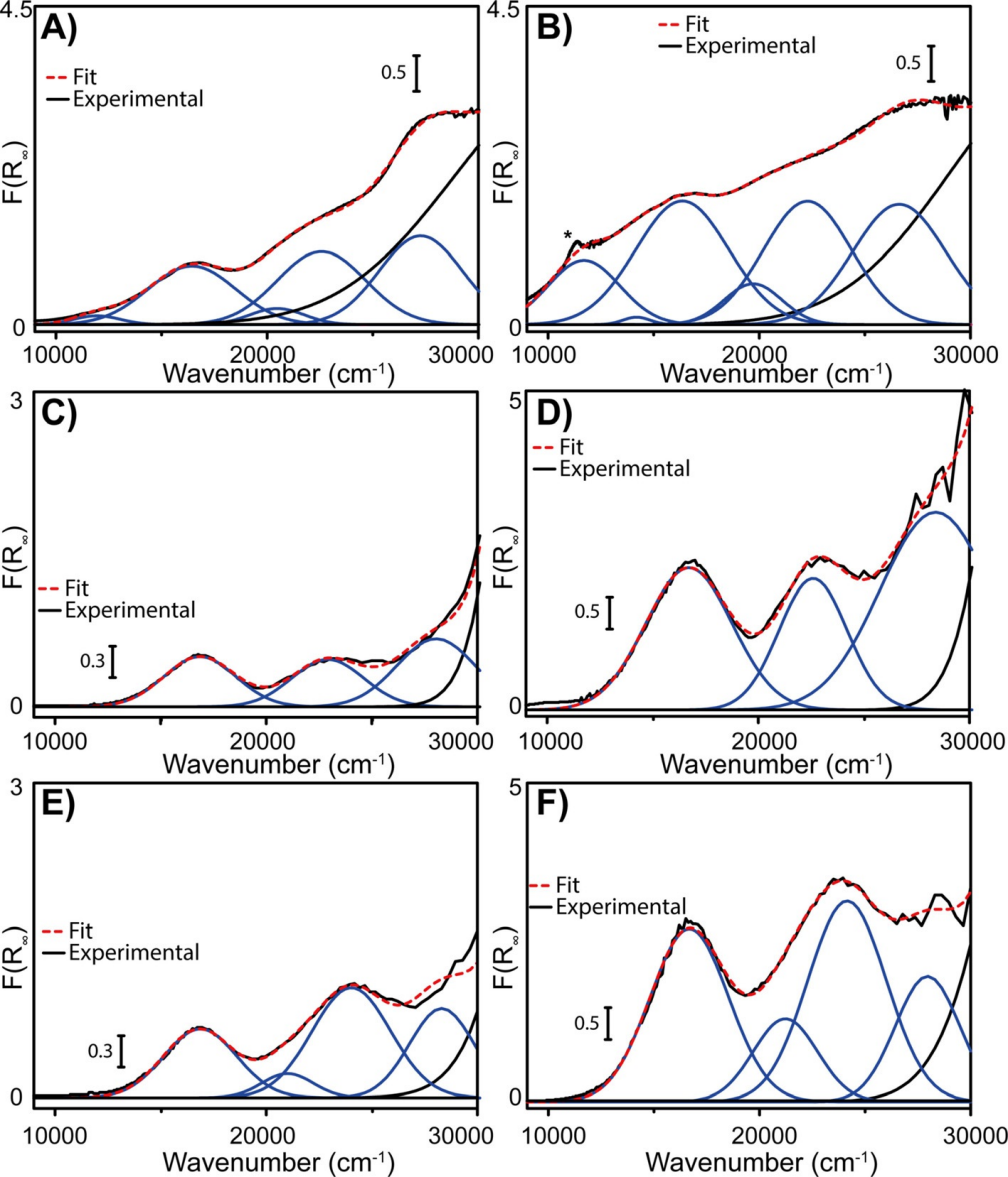
UV/Vis‐NIR diffuse reflectance spectroscopy (DRS) results of the MOFs before and after activation with 100 mol. equiv. Et_2_AlCl. A, B) For MIL‐101(Cr)‐NDC, C, D) for MIL‐53(Cr) and E, F) for HKUST‐1(Cr).

Furthermore, UV/Vis‐NIR DRS served as a tool to investigate the effect of the co‐catalyst on the oxidation state and coordination geometry of the Cr active sites. The spectra of the materials before and after reaction with the organo‐aluminum co‐catalysts are shown in Figure 5. It is worth stating that the starting oxidation states of MIL‐101(Cr)‐NDC and MIL‐53(Cr) are considered to be Cr^3+^, demonstrated by the bands around 16 000 and 22 000 cm^−1^. That of HKUST‐1(Cr) is expected to be Cr^2+^ on basis of the original manuscript, which was also confirmed by the orange color of the material, consistent with the original report. If oxidation of HKUST‐1(Cr) is to occur, a color change from orange to green would be observed, which was excluded. There are small variations from one MOF to another, due to the different electronic structures of the metal centers. The Charge Transfer (CT) bands above 30 000 cm^−1^ have been omitted for all MOFs, due to their extreme intensity that saturates the detector and therefore renders interpretation impossible.

Activation with the co‐catalyst does affect the spectra to some extent. In each case, reacting the MOF materials with the co‐catalyst resulted in a darkening of the powder material, which in the UV/Vis‐NIR DRS spectra is directly related to an overall increase of intensity of these spectra.

With respect to the oxidation state, none of the UV/Vis‐NIR DRS spectra give strong indications for the formation of any oxidation state besides Cr^3+^, unlike the case of previously reported MIL‐101(Cr).^[43]^ This is surprising given the identical Cr trimer for MIL‐101(Cr) and MIL‐101(Cr)‐NDC, with the pore size being the only difference between these two materials. It is worth stating that the 16 000 cm^−1^ band broadens significantly for MIL‐101(Cr)‐NDC after activation with the co‐catalyst, with two possible chemical explanations. First, activation with the organo‐aluminum complex was found to generate a large variety of active sites, naturally, this translates into a variety of UV/Vis‐NIR absorptions and is related to the observed heterogeneous broadening. Secondly, Et_2_AlCl in fact reduces some of the Cr^3+^ sites from the pristine MOFs to Cr^2+^, which are known to absorb in the 8000–12 000 cm^−1^ region. In this case, the exact location is usually determined by the degree of coordinative saturation, and the fact that broadening is observed around 10 500 cm^−1^ infers that if reduction is occurring it is likely that Cr^2+^_Oh_ is formed (i.e., coordinatively more saturated Cr^2+^). With respect to the fitted bands, these only serve to emphasize broadening of the band at 16 000 cm^−1^ band, with its location and FWHM being identical in Figures 5 A and B.[^25^, ^68^, ^69^, ^70^, ^71^]

The UV/Vis‐NIR DRS spectra after activation for both MIL‐53(Cr) (Figure 5 D) and HKUST‐1(Cr) (Figure 5 F) only demonstrate increased intensities of the Cr bands, Cr^3+^ for MIL‐53(Cr) and Cr^2+^ for HKUST‐1(Cr). However, the width and location of these bands remains identical, indicating that activation with the co‐catalyst does not particularly affect the oxidation state of the MOFs.

**Figure 6:**
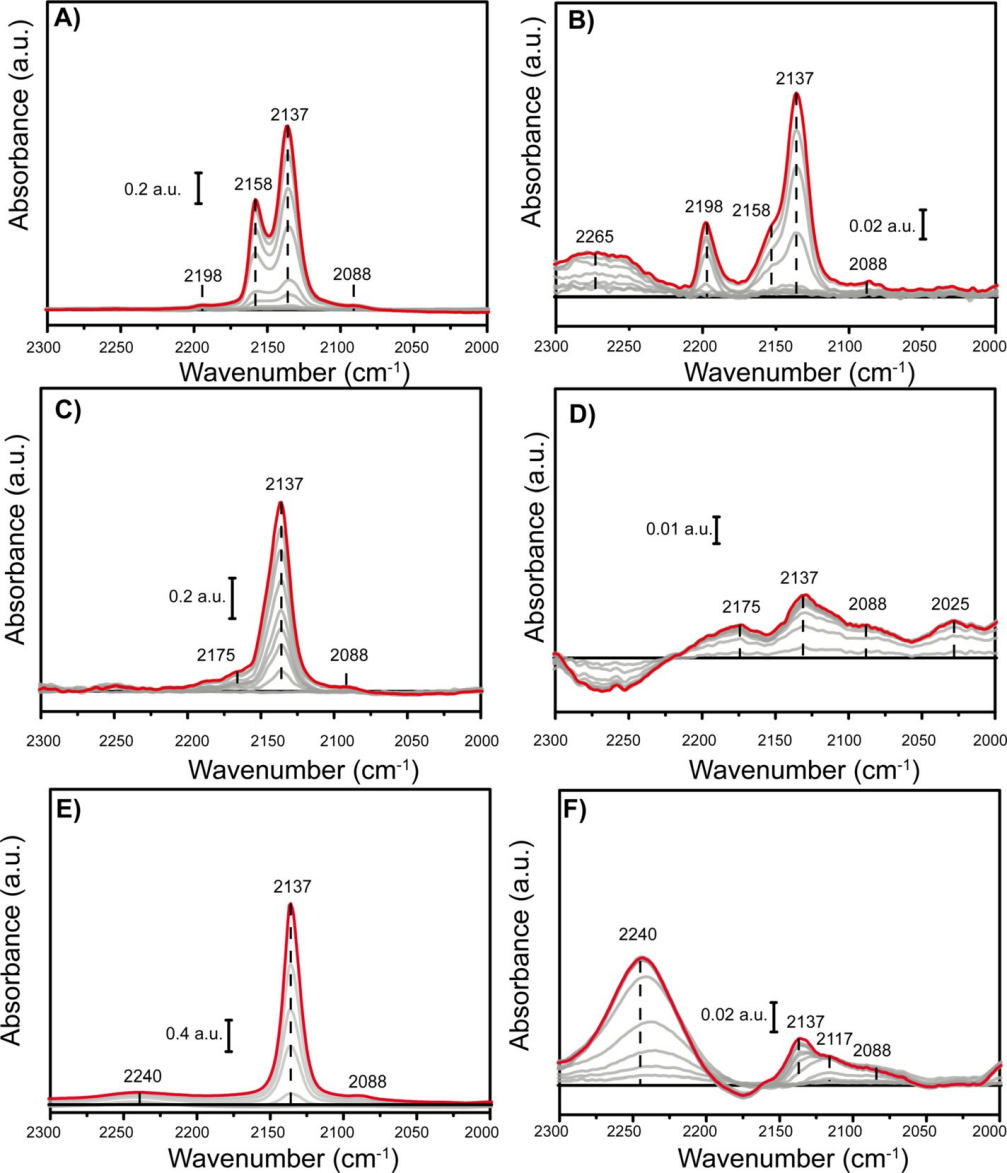
CO probe molecule Fourier transform infrared (FTIR) experiment results of the MOFs before and after activation with 100 mol. equiv. Et_2_AlCl for A, B) MIL‐101(Cr)‐NDC, C, D) MIL‐53(Cr), and E, F) HKUST‐1(Cr).

Next, CO probe molecule FTIR is an excellent tool to probe the Cr site accessibility, while simultaneously providing some information on the related oxidation state.^[72]^ Similarly as in the previous section, CO probe molecule experiments were performed on the pristine MOF materials as well as MOF materials that were activated with 100 mol. equiv. Et_2_AlCl, the results of these experiments are shown in Figure 6.

Figure 6 A and **6B** respectively illustrate the results obtained for MIL‐101(Cr) before and after activation with 100 mol. eq. Et_2_AlCl. It is worth stating that the band at 2137 cm^−1^ can exclusively be attributed to physisorbed CO on the MOF material. The second band, at 2158 cm^−1^ can be attributed to CO coordinated to H_2_O moieties still reminiscent from the hydrothermal synthesis. Additionally, two bands with low intensity at 2198 and 2088 cm^−1^ are present in the spectra, which based on the literature can be attributed to minor amounts of, respectively Lewis acidic (LA) Cr^3+^ sites and extra‐framework Cr (oxidation state 2+ or 3+). With the UV/Vis‐NIR DRS spectra in mind, extra‐framework Cr^3+^ is more likely since no hard evidence for Cr^2+^ in the pristine material was found. Activation with the co‐catalyst, as seen in Figure 6 B, results in a significant decrease of the 2158 cm^−1^ band, likely due to scavenging of the coordinating H_2_O moieties by Et_2_AlCl. Second, the band at 2088 cm^−1^ is still similarly intense as before activation, suggesting that only little additional extra‐framework Cr is formed, with both 2+ and 3+ oxidation states now being viable. However, the significant increase in Cr^3+^ LA species, testified by the 2198 cm^−1^ band, does infer that the heterogeneous broadening in Figure 5 B is predominantly caused by the heterogenization of the Cr^3+^ sites rather than the formation of Cr^2+^ species. Last, a new band emerged at 2265 cm^−1^, which is possibly attributed to CO coordinated to Al^3+^ from the co‐catalyst material which is embedded/reacted with the MOF material and could not be washed away.

MIL‐53(Cr) is defined by the ability to only physisorb CO, with its coordinative saturation being well demonstrated by the lack of any additional bands, with exception of the band at 2088 cm^−1^ attributed to minor amounts of extra‐framework Cr, likely oxidation state 3+, as well as the very small band at 2175 cm^−1^ attributed to LA Cr^3+^. It is worth mentioning that in this case MIL‐53(Cr)‐np (np=narrow‐porous) is likely the phase of MIL‐53(Cr) under investigation. Performing the CO probe molecule FTIR experiments on the activated MIL‐53(Cr) results produces a strongly changed spectrum. First, it appears that the ability to physisorb CO has significantly decreased Now, the relative intensity of the LA Cr^3+^ bands and extra‐framework Cr bands is larger. However, one should take note that the absolute intensities of these bands remain very small. Additionally, one band at 2025 cm^−1^ can now be discerned, which infers the possibility of few‐atom Cr clusters.

Last, the CO probe molecule FTIR spectrum for HKUST‐1(Cr), as shown in Figure 6 E, is defined by only one major signal attributed to the physisorption of CO. This indicates the absence of coordinatively unsaturated sites (CUS) formed after evacuating/drying during the synthesis, suggesting that either DMF molecules from the synthesis or OH molecules from the solvent exchange remain coordinated to the Cr sites, even under the conditions used in this study. Apart from the band at 2137 cm^−1^, only minor bands are observed at 2088 and 2240 cm^−1^, the former is again attributed to extra‐framework Cr^2+^. Performing the same activation procedure with Et_2_AlCl on HKUST‐1(Cr) resulted in a band at 2240 cm^−1^, along with bands at 2117 and 2088 cm^−1^. Due to the unlikeliness of any other oxidation state than 2+, the latter signal is again attributed to extra‐framework Cr^2+^. Interestingly, the 2117 cm^−1^ band falls in the range where normally few‐atom Cr clusters (either oxidation state 2+ or 3+) are observed, which considering the structure of the metal‐node is not an unlikely explanation.^[72]^ In any case, the fact that the intensity of this band is low is a testament to the stability of this MOF, indicating that only a small number of the original linker‐Cr bonds is broken.

In summary, it is clear that the XRD experiments indicate that the more active MOFs are modified to a larger extent. The CO FTIR experiments further corroborate the generation of a variety of active sites, unique to each MOF, which retain the oxidation state of the pristine MOF.

### Estimating collapse

From the X‐ray diffractograms and CO‐FTIR spectra shown above, collapse of the framework may have occurred, affecting the catalytic properties of the MOFs by limiting accessibility of ethylene or chain growth. We used N_2_ physisorption at −196 °C to evaluate the porosity of the MOFs before and after activation with the co‐catalyst.

The respective isotherms of the pristine MIL‐101(Cr)‐NDC, MIL‐53(Cr) and HKUST‐1(Cr) are shown in Figures S12, S14 and S16 with BET surface areas of 1419, 1531 and 1033 m^2^ g^−1^. In any case, Type I isotherms are observed for all the MOFS, confirming their porosity. It is likely that collapse of the MOF is related to a disappearance of the material's porosity as well as a potential change in the observed type of isotherm. While Figures S13, S15 and S17 do confirm that part of the surface area is lost, apparent BET surface areas of 635, 124 and 468 m^2^ g^−1^, indicate that still a significant part of the porosity is retained. The largest drop is observed in case of MIL‐53(Cr), suggesting that in this case porosity might not be correlated to catalytic activity. It is worth stating that drying of the pristine MOFs was performed under dynamic high‐vacuum, while this is supposedly a safe possibility for these MOFs, it is important to consider that vacuum *might* have detrimental effects on the surface area, explaining the slightly lower value compared to those observed in the literature in case of MIL‐101(Cr)‐NDC.[^73^, ^74^]

**Figure 7:**
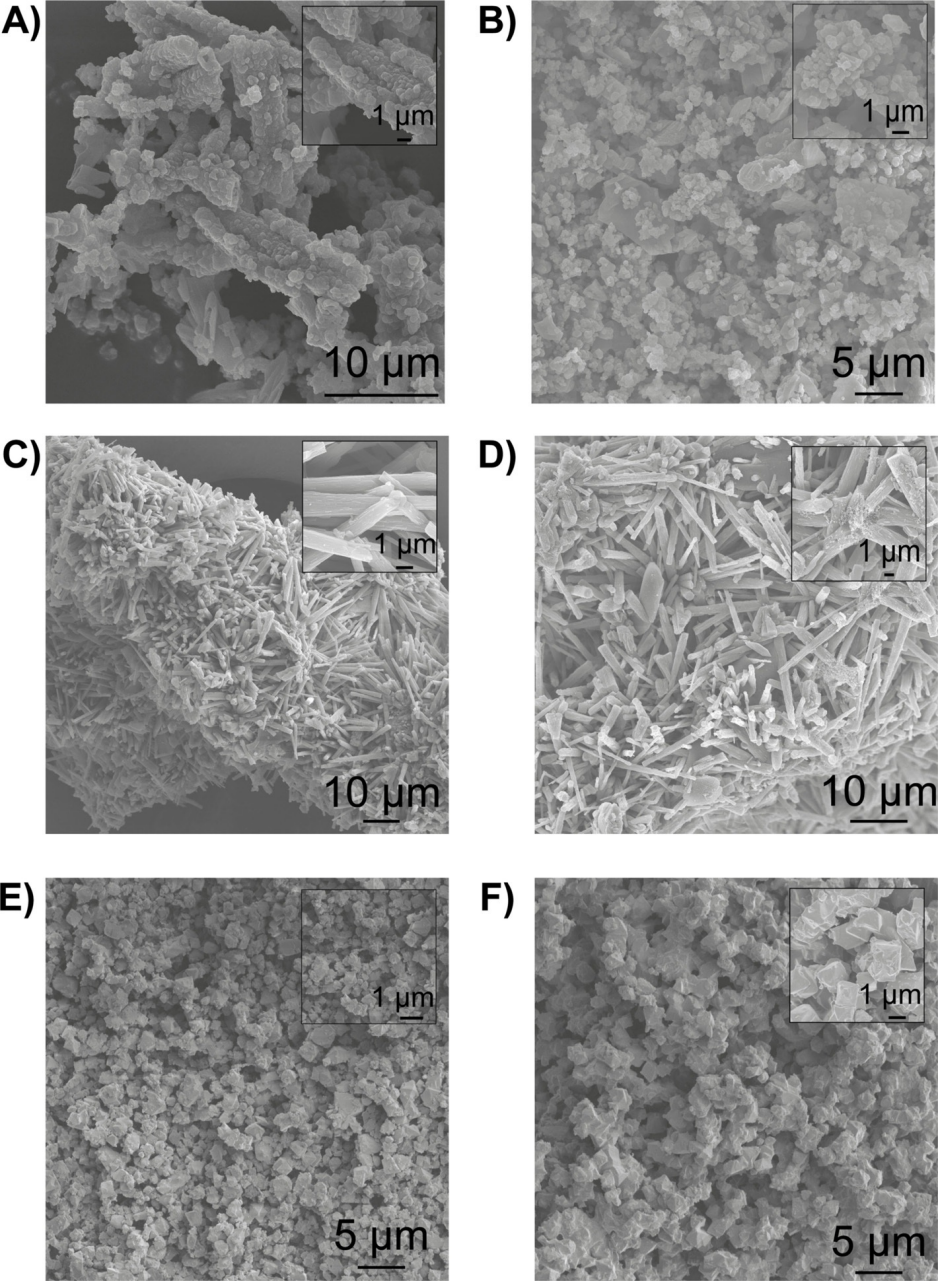
SEM images of the MOFs before and after activation with 100 mol. equiv. Et_2_AlCl for A, B) MIL‐101(Cr)‐NDC, C, D) MIL‐53(Cr) and E, F) HKUST‐1(Cr), respectively.

Second, the morphology of all the MOFs, as shown in Figure 7, has been extensively described in the literature and matches the crystals observed in our case. For MIL‐101(Cr)‐NDC that means nanosized octahedral crystals clustered into larger structures, as shown in Figure 7 A.[^75^, ^76^] For MIL‐53(Cr) a crystalline material is obtained, which consists of exclusively needles (sized 5–10 μm). It is worth stating that MIL‐53(Cr) is the thermodynamic product of MIL‐101(Cr).[^57^, ^77^, ^78^, ^79^] In case of HKUST‐1(Cr), a uniform powder consisting of the octahedral crystals in Figure 7 E is obtained. Again, this is perfectly in line with what is reported in the literature on the morphology of this MOF.[^80^, ^81^, ^82^] It is worth stating that our research does not provide basis to comment on any potential MOF morphology/activity relations, due to the varying metal‐nodes of the MOFs which in that case should have been constant. However, this does warrant further research where the metal‐node structure is kept identical and the particle size/shape is systematically varied.

The morphologies of the activated MOFs are shown in Figures 7 B, D, and F, in which no obvious changes are visible, confirmed by the preserved shapes and sizes of the MOF crystals. It is worth stating that no Al_2_O_3_ is observed in the bright‐field micrograph as a possible by‐product from the activation procedure.

Diffuse reflectance infrared spectroscopy (DRIFTS) was used to investigate whether the characteristic MOF fingerprint bands changed during activation with the co‐catalyst.

**Figure 8:**
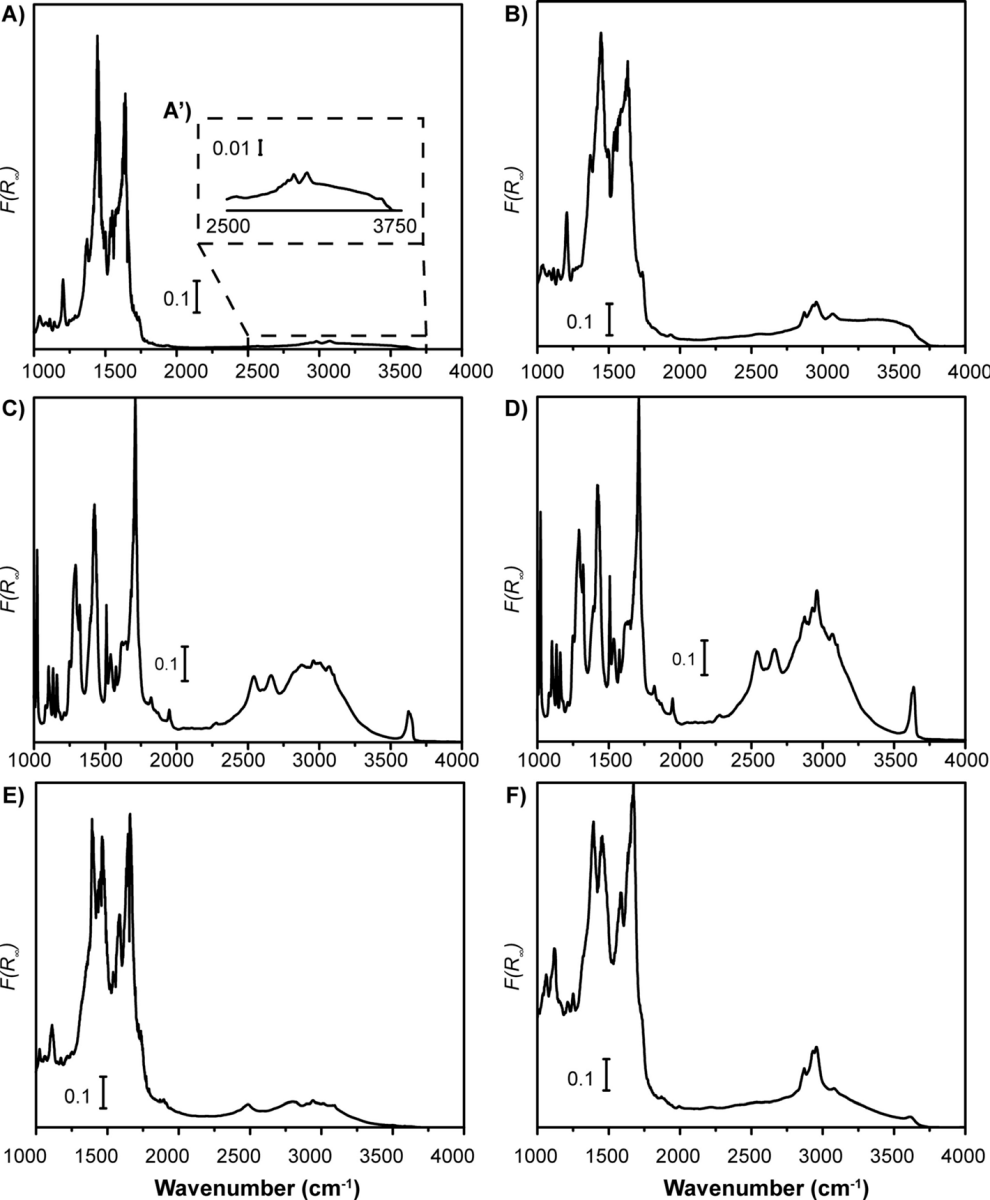
Diffuse reflectance infrared spectroscopy (DRIFTS) spectra of the MOFs before and after activation with 100 mol. equiv. Et_2_AlCl. A, B) for MIL‐101(Cr)‐NDC, C, D) for MIL‐53(Cr) and E, F) for HKUST‐1(Cr).

In the case of MIL‐101(Cr)‐NDC (Figure 8 A) the obtained DRIFTs spectrum is in line with reported spectra. Worth noting, our preparation of MIL‐101(Cr)‐NDC yielded a product without free 2,6‐dicarboxynapthalene, which may potentially block pores as well Cr^3+^ CUS sites, testified by the absence of a *v*(C=O) band at 1700 cm^−1^. Secondly, activation of the MOF, as shown in Figure 8 B, did not decrease the S/N ratio nor did it affect the MOF fingerprint. It is worth stating that newly emerged bands at 2870 and 2950 cm^−1^ can be assigned to alkylation of the MOF by the Et_2_AlCl co‐catalyst, as they correspond to the stretching vibrations of the ethyl groups.

MIL‐53(Cr) (shown in Figure 8 C) is characterized by isolated Cr^3+^_Oh_ metal centers, linked in infinite chains 1D by terephthalic acid with *μ*_2_‐OH groups bridging the individual chains. These *μ*_2_‐OH groups give rise to the characteristic 3600 cm^−1^ *v*(OH) band. Again, only the newly emerged *v*_as/s_(CH_2_) and *v*_as/s_(CH_3_) bands are evident and the persistence of the *μ*_2_‐OH indicates that the overall structure is largely untouched.

HKUST‐1(Cr) behaves very similar, as shown in Figures 8 E and F. The DRIFTS spectrum of pristine HKUST‐1(Cr) testifies to the successful synthesis where no free trimesic acid is observed which might potentially block the pores and/or Cr^3+^ sites. Interestingly, the 1640–1650 cm^−1^ range shows two signals, of which one likely belongs to bound DMF originating from the MOF synthesis. Activation with the co‐catalyst resulted in the disappearance of this signal, related to abstraction of DMF, while alkylation occurred simultaneously.

Despite the significant concern of MOF collapse after activation with the Et_2_AlCl co‐catalyst, the above results show that the chemical bonds comprising the MOF seem to be unaffected. This points to the fact that, in terms of morphology and bonding, the MOF remains intact. While the loss of crystallinity is related to the observed loss in porosity, it is highly important to state that activation with Et_2_AlCl does not result in total collapse of the MOF. This suggests that the resulting materials are partially disordered and porous Cr^3+^ carboxylates with a variety of Cr^3+^ embedded (and potentially extra‐framework Cr^3+^ and Cr^2+^) alkylated sites for MIL‐101(Cr)‐NDC and MIL‐53(Cr), whereas this involves Cr^2+^ sites for HKUST‐1(Cr). From our study, it seems that more generated defects, that is, MIL‐101(Cr)‐NDC and MIL‐53(Cr), is related to higher ethylene polymerization activity. The catalytic activity was related to large *Ð* values, confirming the presence of a variety of active Cr sites. However, further experiments are necessary to disentangle the contribution of each of the solid and/or homogeneous Cr compounds to the MWD. It is also clear that a larger percentage of PE produced over homogeneous Cr complexes leads to more fibrous HDPE, probably due to uncontrolled growth.

## Conclusions

In this work, we have investigated the performance of MIL‐101(Cr)‐NDC, MIL‐53(Cr) and HKUST‐1(Cr) in ethylene polymerization. First, we have found that selecting the appropriate MOF as polymerization platform is of paramount importance for attaining desirable levels of activity with MIL‐53(Cr) being the most active, followed by MIL‐101(Cr)‐NDC and eventually HKUST‐1(Cr). Although the three MOFs demonstrated activity, we found that some of the activity originated from leached Cr complexes.

Secondly, the poly‐ethylenes (PEs) produced over the MOFs demonstrated large *Ð* values as well as varying crystallinities, indicating that ethylene polymerization occurs over a large variety of active sites. Furthermore, selection of the appropriate MOF was critical for templating the final PE morphology, with only MIL‐101(Cr)‐NDC producing favorable PE spheres and HKUST(1)‐Cr demonstrating potential by predominantly producing spheres as well, however with some fibers.

Thirdly, our spectroscopic investigations indeed confirmed that activation of the MOFs results in the generation of a variety of active sites, while retaining the oxidation state of the pristine MOF. Furthermore, we found that the MOFs which were the most modified were also the most active, indicating that proper activation of the MOFs is a prerequisite for ethylene polymerization.

Lastly, it was critical to exclude total collapse of the MOF, which on basis of the GPC and spectroscopic results was a likely event. Activation of the pristine MOFs indeed resulted in decreased BET surface areas (SA), with porous materials (BET SA>400 m^2^ g^−1^) still being the predominant products. Furthermore, activation of the MOF neither affected the MOF morphology or the MOF DRIFTS fingerprint, indicating that activation does not result in cleavage of all the bonds constituting the MOF.

In summary, we have explored three renowned Cr‐based MOFs as ethylene polymerization platforms. In this work, we have shown that selecting the appropriate MOF is critical for the activity, PE properties as well as PE morphologies. More importantly, we have also demonstrated that the active MOF cannot be considered a single‐site heterogenized ethylene polymerization catalyst. We believe that these findings can be helpful for the future development of heterogeneous Cr catalysts as well as Cr‐based MOFs and their applications in the important ethylene polymerization reaction.

## Experimental Section

### Catalyst preparation

The synthesis of the MOFs MIL‐101(Cr)‐NDC,^[51]^ MIL‐53(Cr)^[55]^ and HKUST‐1(Cr)^[58]^ was carried out according to previously published procedures. Details on the preparation can be found in the Supporting Information.

### Ethylene polymerization reactions

In a typical polymerization experiment, an amount equivalent to 0.05 mmol of each MOF was suspended in 15 mL of anhydrous heptane (99.9 % anhydrous, stored over molecular sieves, Sigma–Aldrich) or toluene (99.9 % anhydrous, stored over molecular sieves, Sigma–Aldrich) in a stainless‐steel Parr reactor, together with 100:1 (Al:Cr) molecular equivalents of Et_2_AlCl (97 %, Sigma–Aldrich) in an N_2_ filled glovebox (O_2_<1.5 ppm and H_2_O<0.6 ppm). Subsequently, the reactor was attached to an ethylene gas/vacuum system allowing the evacuation and flushing of the lines before ethylene (Linde AG, 99.9 %) polymerization was performed at a constant pressure of 10 bar and a temperature of 23 °C. The reaction mixture was stirred at 1000 rpm. The reactor was depressurized after 30 min of polymerization and the residual Et_2_AlCl was quenched with 1 m HCl in ethanol. The solid product was copiously washed with 1 m HCl in ethanol, followed by ethanol. The solid material was dried at 70 °C overnight and 3 h under vacuum. The catalyst activity was based on the weight of the obtained polymer products. The polymerization reactions with the filtrates were performed as follows. 0.05 mmol of each MOF was suspended in 10 mL of anhydrous heptane (99.9 % anhydrous, stored over molecular sieves, Sigma–Aldrich), stirred for 15 minutes and subsequently collected by filtration, the filter was washed twice with 2 mL of the heptane and once with 1 mL of the heptane in order to reach the desired diluent volume of 15 mL. Subsequently, ethylene polymerization was performed as described before.

### X‐ray diffraction

The X‐ray diffraction (XRD) patterns were obtained with a Bruker‐AXS D2 Phaser powder X‐ray diffractometer in Bragg–Brentano geometry using Co K*α*=1.78897 Å, operated at 30 kV. The measurements were carried out between 5 and 30 °, using a step‐size of 0.05° and a scan speed of 1 s with a 0.1 mm slit for the source. The activated MOF material was prepared as follows. 100 mg of MOF was distributed over a required number of vials so that each vial contained 0.05 mmol MOF. Subsequently, 15 mL of heptane was added and 100 mol. equiv. of Et_2_AlCl and the mixture was homogenized for 15 minutes before collecting the powder by filtration. Hereafter it was washed thrice with 5 mL pentane. The MOF was carefully dried in air for 5 min before being brought outside and carefully exposed to ambient atmosphere. After this, the materials were measured.

### CO probe molecule Fourier transform infrared spectroscopy

Fourier transform infrared (FTIR) spectroscopy measurements with CO probe molecules were recorded on a PerkinElmer 2000 instrument, in a specially designed cell fitted with CaF_2_ windows. The dried MOF materials were pressed into 5–7 mg wafers inside an N_2_ glovebox (O_2_<1.2 ppm and H_2_O <0.6 ppm). The cell was sealed and connected to the gas/vacuum system. Subsequently, the cell was carefully evacuated to 10^−5^ bar at 25 °C, after which the sample was cooled to liquid N_2_ temperature. A mixture of 10 % CO/ 90 % HE (v/v) was dosed with small increments while measuring FTIR spectra 1 min after each CO dosing to ensure equilibration. Experiments performed on the activated materials were performed as follows: 5–7 mg of the pristine MOF was pressed in a self‐supporting pellet, after which it was carefully suspended for 15 min in a mixture containing 15 mL heptane and 100 mol. eq. Et_2_AlCl. Subsequently it was suspended in 15 mL pentane to wash away the excess Et_2_AlCl. Subsequently, the CO probe molecule experiments were performed as previously described. Extra care was taken to ensure that there were no cracks in the pellet after activation.

### UV/Vis‐NIR diffuse reflectance spectroscopy

UV/Vis‐NIR Diffuse Reflectance Spectroscopy (DRS) measurements were performed using a PerkinElmer Lambda950s spectrophotometer with a Praying Mantis DRS accessory. The measurements were performed in the 40 000‐4000 cm^−1^ region with a 60 ms datapoint scan time and 4 nm spectral resolution. For every measurement, the Praying Mantis DRS was loaded with 10–20 mg of MOF inside an N_2_ filled glovebox (O_2_<1.2 ppm and H_2_O<0.6 ppm). The samples were measured against a Teflon white, measured in the same cell, consisting of 30 μm Teflon beads. The activated MOFs were performed as follows: 0.05 mmol MOF was suspended in 15 mL heptane, to which 100 mol. eq. Et_2_AlCl was added. The mixture was stirred for 10 min, after which the powder was collected by filtration and washed twice with 5 mL heptane. The powder was dried in the atmosphere for an additional 10 min before being measured.

### Inductively coupled plasma–atomic emission spectroscopy

ICP‐OES was used for chemical analysis. 0.05 mmol of the pristine MOF was weighed and suspended in 15 mL heptane to which 100 mol. eq. Et_2_AlCl was added. Subsequently, the liquid was collected by filtration and the filter was washed twice with 5 mL heptane. Subsequently, the diluents were removed by slow evaporation. The residue was then dissolved in a minimal amount of aqua regia before being diluted to the same pH as a 5 % HNO_3_ solution. 10 mL of the diluted samples were taken for measurements on a PerkinElmer Optima 8300 and an average of three samples was used. Cr (267.7, 205.6 and 283.6 nm) were measured and referenced to 0, 0.2, 0.4, 0.6, 0.8 and 1.0 mg L^−1^ were prepared of all the metals.

### Scanning electron microscopy

SEM measurements were carried out with a FEI Helios NanoLab G3 UC (FEI Company) instrument equipped with a Silicon Drift Detector (SDD) at 10.0 kV acceleration voltage and a 0.10 nA current. The samples were dispersed on an aluminum SEM stub with a carbon sticker and were subsequently coated with a Pt layer.

### Differential scanning calorimetry

DSC was performed on a TA Instruments DSC Q20 with 1–2 mg of the nascent material. Each sample was heated from −40 °C to 200 °C at a rate of 10 °C min^−1^ after which it was held isothermally at 200 °C to erase thermal history of the PE. Subsequently the cooling cycle was initiated to −40 °C at a rate of 10 °C min^−1^ followed by an additional heating cycle to 200 °C at a rate of 10 °C min^−1^. The crystallinities of the polyethylene materials were determined assuming *ΔH_m_* ^*0*^=293 J g^−1^ for 100 % crystalline ultrahigh‐molecular‐weight polyethylene (UHMWPE).

### Gel permeation chromatography–size exclusion chromatography

Gel permeation chromatography‐size exclusion chromatography (GPC‐SEC) was carried out on a Polymer Laboratories PL‐GPC220 instrument in 1,2,4‐trichlorobenzene at 160 °C, equipped with a PL BV‐400 refractive index detector. The column set consisted of three Polymer Laboratories 13 μm PLgel Olexis 300×7.5 mm columns, and the calibration was performed with linear polyethylene (PE) and polypropylene (PP) standards.

### N_2_ physisorption

N_2_ adsorption measurements for MIL‐101(Cr)‐NDC and MIL‐53(Cr) were measured at 77 K on a Micromeritics TriStar 3000 instrument. Prior to all measurements, samples were dried at 150 °C under dynamic vacuum. Specific surface areas (SSAs) were calculated using the multipoint BET method (0.05<*p*/*p*_0_<0.25). N_2_ measurements for HKUST‐1(Cr) were performed as follows: high‐resolution low‐pressure adsorption measurements were measured on a Micromeretics ASAP2010 gas adsorption analyzer equipped with additional 1 mmHg and 10 mmHg pressure transducers. A relative pressure range from *p*/*p*_0_=10^−7^ to 0.99 has been applied. Before the actual measurements on this apparatus, the samples were degassed for 16 h at 150 °C. Specific surface areas (SSAs) were calculated using the multipoint BET method (0.05<*p*/*p*_0_<0.25).

## Conflict of interest

The authors declare no conflict of interest.

## Supporting information

As a service to our authors and readers, this journal provides supporting information supplied by the authors. Such materials are peer reviewed and may be re‐organized for online delivery, but are not copy‐edited or typeset. Technical support issues arising from supporting information (other than missing files) should be addressed to the authors.

Supplementary
