# Supplementary material for: Influence of Pore Structure and Metal‐Node Geometry on the Polymerization of Ethylene over Cr‐Based Metal–Organic Frameworks
Source: Chemistry. 2021 Mar 1;27(18):5769–81. doi: 10.1002/chem.202005308 (PMC8049024; doi:10.1002/chem.202005308)
Supplement: Supplementary file 1 — Supplementary [file CHEM-27-5769-s001.pdf]

Chemistry–A European Journal

Supporting Information

Influence of Pore Structure and Metal-Node Geometry on the Polymerization of Ethylene over Cr-Based Metal–Organic Frameworks

Maarten K. Jongkind, Miguel Rivera-Torrente, Nikolaos Nikolopoulos, and Bert M. Weckhuysen*[a]

Table of Contents

1. **Synthesis of the Metal-Organic Frameworks**
2. **Thermogravimetric Analysis (TGA) of the pristine MOF powders**
3. **Differential Scanning Calorimetry (DSC) of the nascent PE materials**
4. **N_2 Physisorption of the Pristine and Activated MOFs**

1. Synthesis of the Metal-Organic Frameworks

Materials and Methods: dry glassware was kept in an oven at 120 °C prior to use and the Schlenk experiments were carried out under Ar using dry flasks and joints. In the case of HKUST-1(Cr), the solvents: DMF (anhydrous, 99.9%, Sigma-Aldrich) and MeOH (anhydrous, 99.8%, Sigma-Aldrich) were kept in 3 Å mol sieves and degassed by sparging Ar for 2 h prior to use.

Synthesis of MIL-101(Cr)-NDC: 2,6-naphthalenedicarboxylic acid (430 mg, 2 mmol, Sigma-Aldrich, 95%), $Cr(NO_3)_3 \cdot 9H_2O$ (800 mg, 2 mmol, Sigma-Aldrich, 98%), glacial acetic acid (23.8 mL, 4 mmol, Merck KgaA, +99%), and deionized H_2O (9,54 mL) were introduced into a Teflon liner inserted into a stainless steel Parr autoclave and stirred for 20 min. The autoclave was then heated to 200 °C over the course of 3 h, then kept at 200 °C for 5 h, and finally cooled to room temperature over 3 h. The reaction product MIL-101(Cr)-NDC was recovered by centrifugation as a green powder, washed with water (50 mL), ethanol (50 mL) and soaked in ethanol at 60 °C for 24 h under stirring in a round bottom flask. Then, the powder was recovered and dried *in vacuo* at 150 °C for 24 h and subsequently introduced into a glovebox until further use.

Synthesis of HKUST-1(Cr): $Cr(CO)_6$ (8.2 g, 10 mmol), trimesic acid (H_3BTC, 1.2 g, 6 mmol) and degassed (purged with Ar for 2 h and dried in 3 Å mol sieves) anhydrous DMF (80 mL) were introduced into a dry 250 mL Schlenk flask under Ar atmosphere. The mixture was refluxed (150 °C) for 3 days under Ar flow and then cooled to 25 RT. The violet suspended powder was isolated by Schlenk filtration and rinsed three times with anhydrous oxygen-free DMF. Then, the DMF was removed with a canula and the product soaked with 100 mL of degassed methanol. After 24 h, the methanol was decanted to afford a dark orange solid, and the product dried under vacuum at room temperature for 24 h and at 100 °C for 24 h before being introduced in the glovebox as an orange powder.

Synthesis of MIL-53(Cr): a mixture of chromium(III) nitrate $Cr(NO_3)_3 \cdot 9H_2O$ (Sigma-Aldrich, 98%), terephthalic acid (Sigma-Aldrich, 98%), hydrofluoric acid (HF) (Sigma-Aldrich, solution 48-51% v/v), and H_2O (20 mL) in the molar ratio 1:1:1:280; was introduced in a Teflon liner within a stainless steel Parr autoclave and introduced into an oven at 220 °C for 3 days, then cooled naturally to room temperature. A purple powder with long white needles of terephthalic acid was recovered by centrifugation. Then, the mixture was soaked in DMF at 90 °C under stirring for 24 h to dissolve the linker, then the purified MOF powder recovered. Thereafter, it was washed with EtOH (VWR International, technical, 98%) twice then soaked in DMF at 90 °C under stirring for 24 h, subsequently in ethanol at 60 °C for 24 h, recovered by centrifugation, dried in air at room temperature and at vacuum at 150 °C for 12h.

2. Thermogravimetric Analysis Experiments of the pristine MOF powders

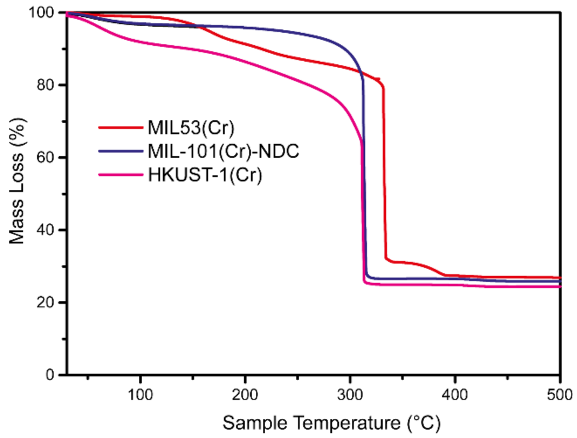

Figure S1. Mass-loss traces obtained from the Thermogravimetric Analysis (TGA) experiments, performed in O_2 in atmosphere, on the pristine Metal Organic Framework (MOF) powders.

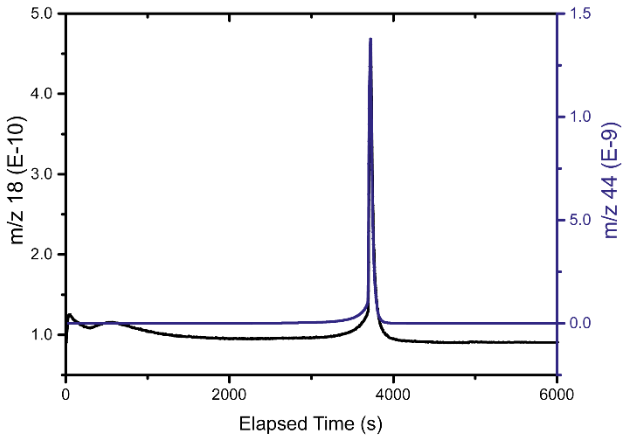

Figure S2. Mass Spectrometry (MS) signal traces for H_2O (black) and CO_2 (blue) obtained during the Thermogravimetric Analysis (TGA) experiments for MIL-101(Cr)-NDC.

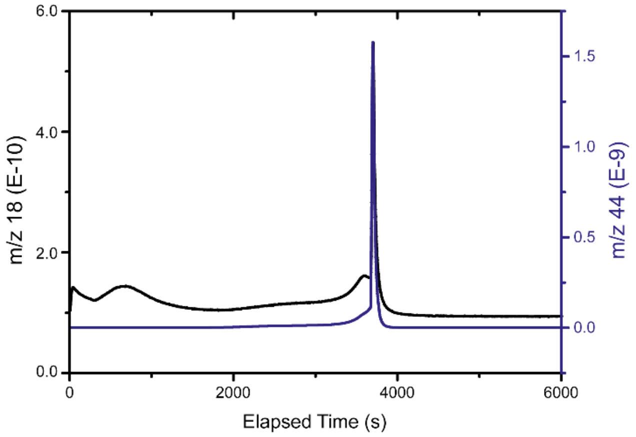

Figure S3. Mass Spectrometry (MS) signal traces for H_2O (black) and CO_2 (blue) obtained during the Thermogravimetric Analysis (TGA) experiments for HKUST-1(Cr)

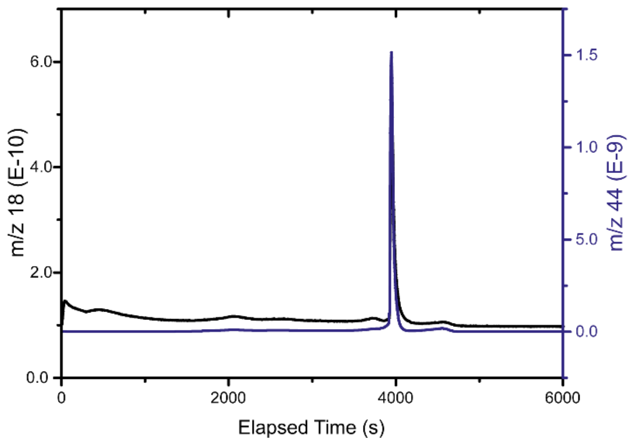

Figure S4. Mass Spectrometry (MS) signal traces for H_2O (black) and CO_2 (blue) obtained during the Thermogravimetric Analysis (TGA) experiments for MIL-53(Cr)

3. Differential Scanning Calorimetry of the nascent polyethylene powders

In all traces, positive curves represent the second melting cycle (endothermic heat flow, ΔH > 0). Negative curves (exothermic heat flow, ΔH < 0) reflects the crystallization temperature. The melting enthalpy per gram of polymer was calculated by integrating the melting peak.

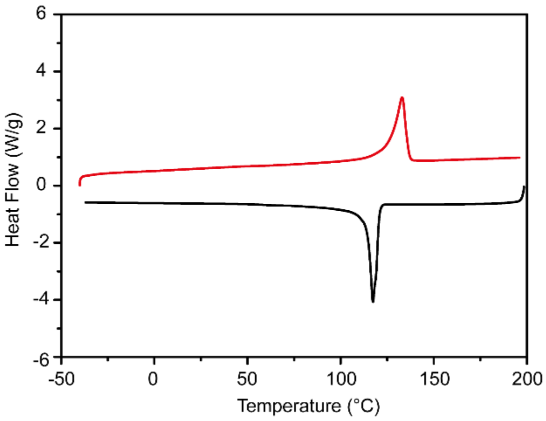

Figure S6. Differential Scanning Calorimetry (DSC) curves for the with MIL-101(Cr)-NDC produced polyethylene product.

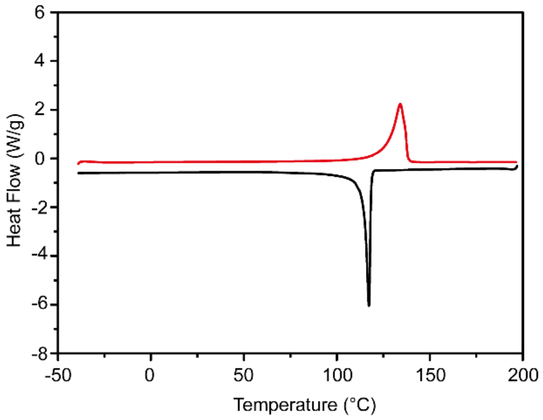

Figure S7. Differential Scanning Calorimetry (DSC) curves for the with the filtrate of MIL-101(Cr)-NDC produced polyethylene product.

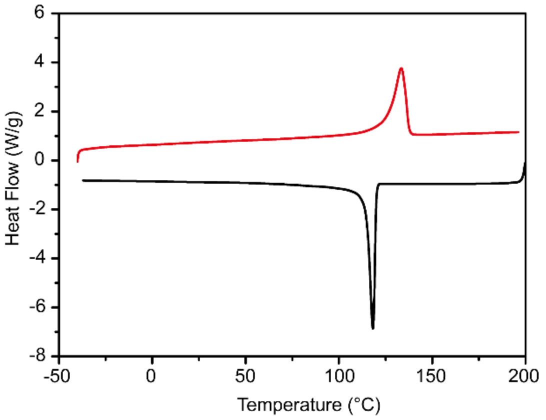

Figure S8. Differential Scanning Calorimetry (DSC) curves for the with MIL-53(Cr) produced polyethylene product.

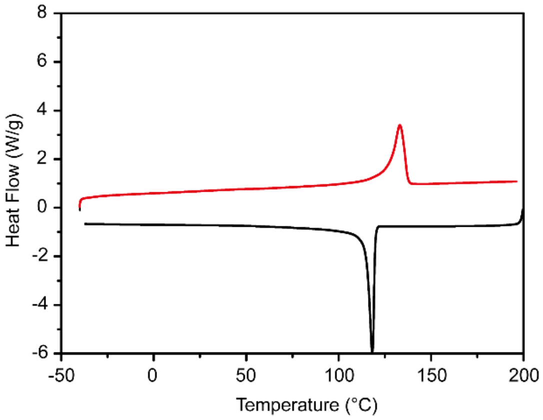

Figure S9. Differential Scanning Calorimetry (DSC) curves for the with the filtrate of MIL-53(Cr) produced polyethylene product.

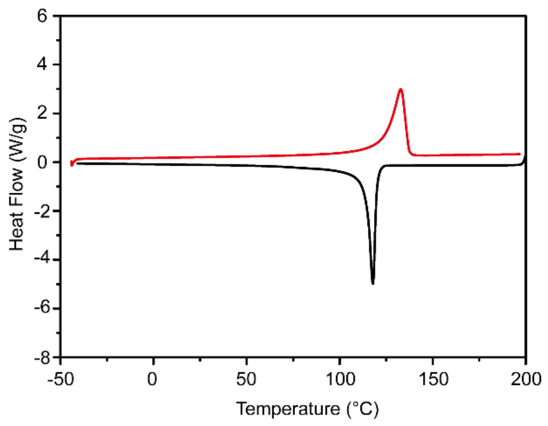

Figure S10. Differential Scanning Calorimetry (DSC) curves for the with HKUST-1(Cr) produced polyethylene product.

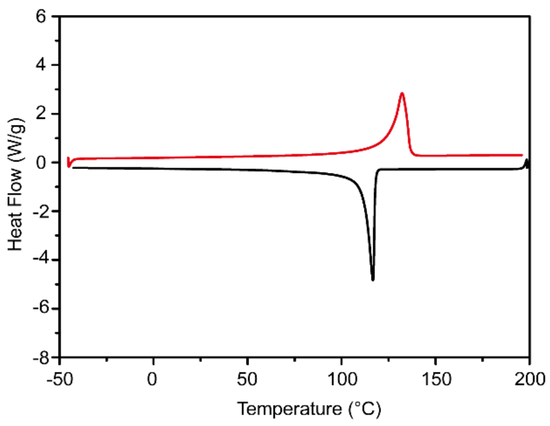

Figure S11. Differential Scanning Calorimetry (DSC) curves for the with the filtrate of HKUST-1(Cr) produced polyethylene product.

4. N_2 Physisorption results of the pristine and activated MOFs

In the isotherms below, the blue curve represents the adsorption curve, red represents the desorption curve.

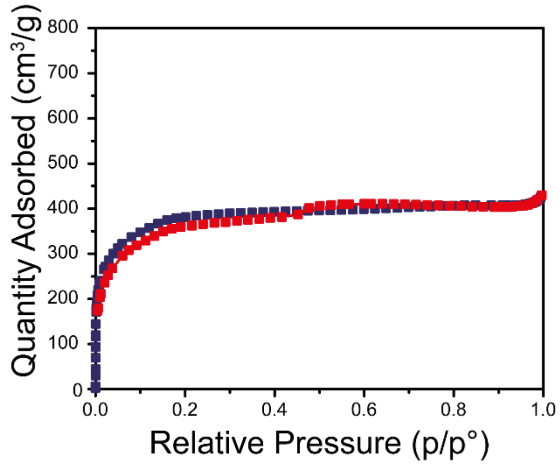

Figure S12. Isotherm obtained from N_2 Physisorption experiments for Pristine MIL-101(Cr)-NDC.

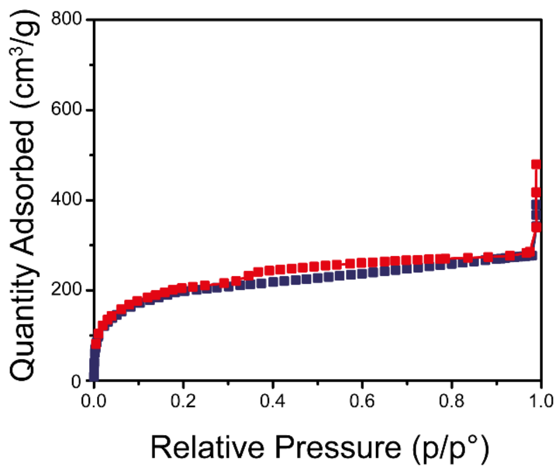

Figure S13. Isotherm obtained from N_2 Physisorption experiments for MIL-101(Cr)-NDC activated with 100 molecular equivalents Et_2AlCl.

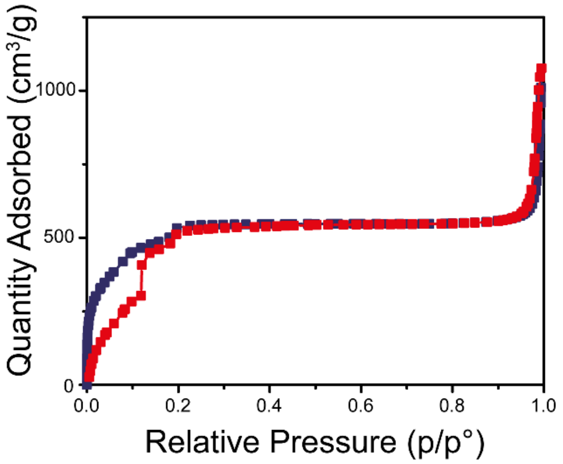

Figure S14. Isotherm obtained from N_2 Physisorption experiments for Pristine MIL-53(Cr).

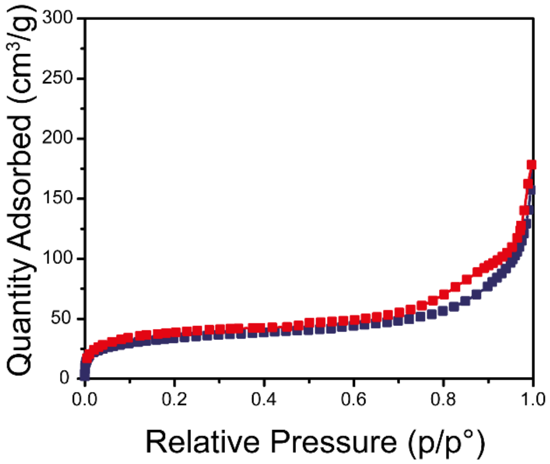

Figure S15. Isotherm obtained from N_2 Physisorption experiments for MIL-53(Cr) activated with 100 molecular equivalents Et_2AlCl.

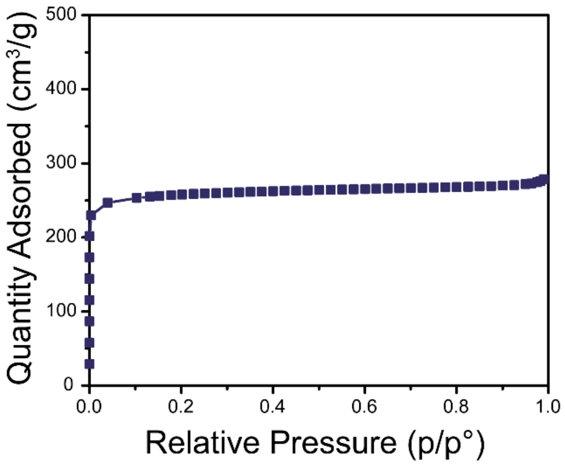

Figure S16. Isotherm (adsorption) obtained from N_2 Physisorption experiments for Pristine HKUST-1(Cr).

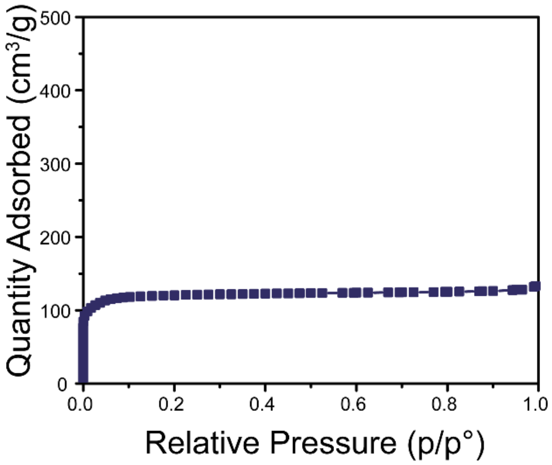

Figure S17. Isotherm (adsorption) obtained from N_2 Physisorption experiments for activated HKUST-1(Cr).
